# Receiving Peer Evaluation in Team-Based Learning: A Descriptive Phenomenological Study of Preclinical Medical Students

**DOI:** 10.3390/bs16091462

**Published:** 2026-08-23

**Authors:** Bomyee Lee, Su Jin Chae

**Affiliations:** Department of Medical Education, University of Ulsan College of Medicine, Ulsan 44033, Republic of Korea; leebomyee@ulsan.ac.kr

**Keywords:** team-based learning, peer evaluation, peer feedback, feedback literacy, self-regulated learning, emotion, medical students, qualitative research

## Abstract

Learning from feedback depends not only on the feedback provided but also on how learners interpret, evaluate, and respond to it. This descriptive phenomenological study explored how preclinical medical students experienced receiving peer evaluation in a team-based learning (TBL) course. Forty-one reflective journals written by first-year medical students (27 men, 14 women) after receiving anonymous peer feedback were analyzed using Colaizzi’s seven-step descriptive phenomenological method. Five interrelated themes emerged: preparing to contribute, encountering oneself through peers’ eyes, making sense of emotional responses, transforming feedback into future practice, and becoming a responsible evaluator. Students described peer feedback as a social mirror that revealed differences between their self-perceptions and those of their peers. They reported a range of emotional responses and also reflected on the credibility and relevance of the feedback they received. Many students expressed intentions for future participation, and some reflected on their own responsibilities as evaluators. Taken together, the accounts described self-reflection, emotional appraisal, and intentions for future participation after peer evaluation. The findings suggest that peer evaluation accompanied by open-ended reflection may provide a useful context for examining how students experience and make sense of feedback. Whether such reflection contributes to subsequent changes in behavior, feedback literacy, self-regulated learning, or professionalism requires further study.

## 1. Introduction

How learners interpret and respond to feedback is an important concern in the psychology of learning. Collaborative professional education provides repeated opportunities for learners to give, receive, and respond to feedback within ongoing team interactions. Team-based learning (TBL) has become a widely used instructional approach in undergraduate medical education because it promotes active learning, collaboration, and accountability (Michaelsen et al., 2004; Parmelee et al., 2012). Peer evaluation is commonly incorporated into TBL to provide information about individual contributions to team learning (Cestone et al., 2008).

Despite its central role within TBL, peer evaluation remains one of the most controversial and inconsistently implemented components of the curriculum. Numerous medical schools either minimize its influence on assessment or avoid rigorous implementation altogether because students often experience discomfort when evaluating peers, worry about damaging interpersonal relationships, or lack confidence in their ability to provide constructive comments (Burgess et al., 2021; Lerchenfeldt et al., 2019). Consequently, educational efforts have largely focused on improving the quality of feedback students provide by developing scoring rubrics, feedback training, and structured evaluation formats (Lerchenfeldt et al., 2019).

Existing research reflects this emphasis. Studies examining peer evaluation in TBL have predominantly investigated the giving side of feedback, exploring the accuracy, quality, and acceptability of comments written by students. For example, Burgess et al. demonstrated that students were generally able to provide positive feedback but were considerably less comfortable offering constructive criticism, suggesting that limited feedback literacy constrained the educational value of peer evaluation (Burgess et al., 2021). Similarly, Lerchenfeldt and colleagues reported that medical students and residents regarded peer feedback as educationally valuable; however, they identified substantial challenges related to feedback delivery, evaluator preparation, and implementation (Lerchenfeldt et al., 2023). Collectively, these studies have improved our understanding of how students generate peer feedback and how educational programs might enhance its quality.

However, the processes that unfold after feedback is received remain comparatively underexplored. Feedback does not improve learning simply because it is provided. Rather, its educational value depends on how learners interpret, evaluate, accept, emotionally respond to, and ultimately use the information to guide future performance (Winstone et al., 2017). Two students may receive identical feedback yet derive entirely different educational benefits depending on how they make sense of the comments they receive. From this perspective, the educational impact of peer evaluation lies in the quality of feedback itself and in the learners’ capacity to transform feedback into meaningful action.

This learner-centered perspective is reflected in the concept of student feedback literacy, introduced by Carless and Boud (Carless & Boud, 2018). Feedback literacy describes learners’ capacity to appreciate feedback as an opportunity for learning, make informed judgments about performance, manage the emotional responses elicited by evaluation, and translate feedback into future improvement. Importantly, feedback literacy conceptualizes learners not as passive recipients of information but as active participants who interpret, negotiate, and act upon feedback within ongoing learning processes. The model therefore shifts attention away from feedback as information toward feedback as a process of meaning-making (Carless & Boud, 2018; Molloy et al., 2020).

A complementary perspective is offered by theories of self-regulated learning. Zimmerman’s model conceptualizes learning as a cyclical process consisting of forethought, performance, and self-reflection (Zimmerman, 2002). Feedback received after performance becomes educationally valuable only when learners engage in reflective interpretation that subsequently informs future planning and behavioral adjustment. Likewise, Boud and Molloy’s concept of sustainable feedback argues that feedback should not be viewed as a one-way transmission of expert judgment, but as an ongoing dialogue through which learners progressively develop the capability to evaluate themselves and others (Boud & Molloy, 2013). Together, these theoretical perspectives draw attention to the active role of learners in interpreting and responding to feedback.

Reflective writing completed after receiving peer evaluation offers an opportunity to examine how learners describe and make sense of feedback close to the time it is received. Compared with delayed retrospective accounts, such reflections may provide access to students’ initial interpretations, emotional responses, judgments about feedback, and intentions for future participation. However, because reflective writing itself may shape how experience is articulated, the present study treats the journals as accounts of students’ post-feedback experience rather than as direct evidence of a developmental process.

Although qualitative studies have explored students’ perceptions of peer feedback, few have described, from a phenomenological perspective, how medical students experience peer evaluation immediately after receiving feedback in TBL (Lerchenfeldt et al., 2019, 2023). The present study focuses on this immediate post-feedback experience. Feedback literacy and self-regulated learning were used after the descriptive analysis to inform the Discussion; they were not used as a priori coding frameworks.

Accordingly, this study adopted a descriptive phenomenological approach to explore the lived experience of receiving peer evaluation among preclinical medical students participating in TBL. Specifically, we sought to examine (1) how students cognitively and emotionally experienced receiving peer evaluation, (2) how they interpreted and made sense of those experiences, and (3) what intentions they expressed for future learning and participation. By focusing on students’ immediate post-feedback experiences, this study provides a descriptive account of how preclinical medical students experienced and made sense of peer evaluation in TBL.

## 2. Materials and Methods

### 2.1. Study Design

This study employed a descriptive phenomenological methodology to explore the lived experience of receiving peer evaluation among preclinical medical students participating in TBL. Descriptive phenomenology aims to describe a phenomenon as experienced by participants while minimizing the imposition of researchers’ prior assumptions. Colaizzi’s method was selected because its systematic sequence from significant statements to formulated meanings, theme clusters, and an exhaustive description was consistent with the study aim of describing the shared experience represented in the reflective journals (Colaizzi, 1978; Morrow et al., 2015).

The research team consisted of two medical education researchers. Neither researcher was involved in teaching or assessing the Introduction to Clinical Medicine (ICM) course. Peer-evaluation scores and the content of the reflective journals were not included in students’ course grades. The researchers’ prior experiences with TBL, peer evaluation, and qualitative research were considered during the analytic process.

Before formal analysis, the researchers discussed assumptions related to TBL, peer evaluation, and feedback and recorded these reflections in analytic notes. The phenomenological analysis was conducted inductively from the reflective journals. Feedback literacy and self-regulated learning were not used as coding categories or as a framework for theme development; they were introduced after the descriptive analysis to inform the Discussion. Reporting was guided by the Standards for Reporting Qualitative Research (SRQR) (O’Brien et al., 2014).

### 2.2. Educational Context

The study was conducted in the ICM course at the University of Ulsan College of Medicine from 21 February to 7 March 2022, at the beginning of the first preclinical year. The ICM course uses TBL as its primary instructional strategy and is designed to foster collaborative learning, clinical reasoning, communication, and professionalism during the preclinical curriculum.

Students worked in eight fixed teams of five to six members, newly constituted for this course, and completed two TBL sessions over the 2-week instructional block; teams had therefore worked together for approximately two weeks before the peer evaluation. Peer evaluation was conducted once, at the end of the block. Each student anonymously evaluated all other members of the team (four or five peers) by distributing a fixed pool of points such that the average score per teammate was 10 points. Each student assigned a score of 9 or lower to exactly one teammate and a score of 11 or higher to exactly one teammate, with written justification for the highest and lowest scores. Ratings were given in three domains: preparedness, team contribution, and consideration and respect. Scores and anonymous comments were compiled into a confidential individual feedback report. After reviewing the report, students responded to one open-ended instruction: “Please write freely about your thoughts and feelings after reviewing your peer evaluation results.” Students completed the journal directly after reviewing their feedback report; the exact writing time was not recorded. The peer-evaluation form is provided in Supplementary Material S1.

### 2.3. Participants and Data Collection

Participants were first-year medical students enrolled in the ICM course. The phenomenon of interest was the lived experience of receiving peer evaluation in TBL, as described by students in reflective journals completed after reviewing their feedback.

Forty-one reflective journals, written by 27 male and 14 female students, comprised the final dataset. Journal length was not prescribed and varied across students. All 41 journals contained material related to the experience of receiving peer evaluation and were included. Because the study analyzed the complete available cohort, recruitment and data collection were not guided by saturation. The journals were anonymized before analysis, and demographic characteristics were not linked to individual texts. All analytic steps were conducted using the original Korean journals. Quotations selected for publication were translated into English and checked against the Korean source text to preserve meaning; formal back-translation was not used because the analysis was not conducted on translated data.

### 2.4. Data Analysis

Data were analyzed using Colaizzi’s seven-step descriptive phenomenological method (Colaizzi, 1978; Morrow et al., 2015). First, all reflective journals were read repeatedly to achieve immersion in the data and to develop an overall understanding of participants’ experiences. Second, significant statements directly related to receiving peer evaluation were identified and extracted. Third, formulated meanings were developed from each significant statement while remaining closely grounded in participants’ original expressions. Fourth, formulated meanings demonstrating conceptual similarity were clustered into themes through iterative comparison and discussion. Fifth, the resulting themes were integrated into an exhaustive description of the phenomenon. Sixth, the exhaustive description was synthesized into the fundamental structure of the lived experience of receiving peer evaluation.

Two researchers independently reviewed the data during the initial analytic stages and then compared formulated meanings and candidate themes. When interpretations differed, the researchers returned to the original Korean journal entries and discussed the passages in context until agreement was reached. Analytic decisions and revisions to the developing theme structure were documented during the analysis.

Participant validation, the seventh step in Colaizzi’s original procedure, was not conducted because the study used archived reflective journals from a completed course. This limits our ability to determine whether participants would recognize the synthesized description as reflecting their experience. Investigator discussion and documentation of analytic decisions were used to support credibility, but these procedures do not remove this limitation.

Consistent with the aims of descriptive phenomenology, findings are characterized using approximate qualitative quantifiers (e.g., ‘nearly all’, ‘most’, ‘many’, ‘several’, ‘a few’) rather than frequency counts. These expressions are intended to convey the breadth of the dataset and should not be interpreted as indicators of prevalence or statistical importance.

### 2.5. Trustworthiness

Trustworthiness was established according to Lincoln and Guba’s criteria (Lincoln & Guba, 1985). Credibility was enhanced through investigator triangulation, prolonged engagement with the data, iterative analytic discussions, and reflexive memo writing. Dependability was supported through maintenance of a detailed audit trail documenting analytic decisions and theme development. Confirmability was strengthened through phenomenological bracketing and continual reflection on researchers’ assumptions during interpretation. Transferability was facilitated by providing a rich description of the educational context, participants, and analytic procedures, enabling readers to judge the applicability of the findings to other educational settings.

### 2.6. Ethical Considerations

This study analyzed reflective journals written by students after receiving peer feedback as part of routine educational activities. Because the study involved secondary analysis of de-identified data, it was granted exemption from review by the Institutional Review Board of Asan Medical Center (No. 2024-0429). All identifying information was removed before analysis, and access to the anonymized dataset was restricted to the research team throughout the study.

## 3. Results

### 3.1. Overview

Analysis of the 41 reflective journals identified five interrelated themes: preparing to contribute, encountering oneself through peers’ eyes, making sense of emotional responses, transforming feedback into future practice, and becoming a responsible evaluator. Table 1 provides a brief overview of the five themes and one illustrative quotation for each. The following sections present the themes in greater detail with additional quotations and description. Participant quotations are presented in italics.

### 3.2. Theme 1: Preparing to Contribute

Preparation was experienced as considerably more than completing assigned pre-class work. Participants consistently described preparation as becoming ready to make a meaningful contribution to their team’s learning. Students described adequate preparation in relation to greater confidence, less hesitation during discussion, and their perceived responsibility to contribute to the team. Rather than representing an individual academic activity, preparation acquired relational meaning because it determined whether students could actively support collaborative learning.

*I realized that the extent to which I prepared before class determined how actively I could contribute to my team and how much I ultimately learned.*

This quotation demonstrates that preparation was interpreted as the foundation for collaborative engagement rather than simply academic readiness. Several participants similarly explained that careful preparation allowed them to articulate their reasoning more confidently and participate more actively in the discussion.

*When there were pre-class materials, I deliberately read them several times and thought them through. Because of this preparation, I could present my opinions more confidently in class.*

Preparation was also closely associated with accountability toward peers. Students frequently described their concern about becoming a burden to the group when they had not prepared sufficiently. In contrast, they expressed satisfaction when their preparation enabled them to answer questions, stimulate discussion, or help teammates understand difficult concepts. In these reflections, preparation was described as a responsibility toward teammates as well as an individual learning activity.

*I tried to prepare thoroughly both for myself and to avoid causing difficulty for my teammates.*

Not all students described the same degree of preparedness or confidence. Some acknowledged that inadequate preparation was associated with silence, reluctance to speak, or reduced contribution during discussion.

*When I was not sufficiently prepared, I could not trust my own opinions and therefore could not participate actively in discussion.*

Overall, participants described preparation in relation to their readiness to contribute to team learning as well as their own learning.

### 3.3. Theme 2: Encountering Oneself Through Peers’ Eyes

Participants consistently described peer evaluation as an opportunity to encounter themselves through perspectives that had previously remained invisible. Rather than merely confirming existing self-perceptions, feedback from teammates frequently challenged participants’ assumptions about their own participation, communication, and contribution during TBL. This discrepancy between intention and external perception prompted reflection on their own participation.

*I had thought that respecting others and following their opinions was the basic attitude of teamwork. Looking back, I had not realized that this could appear to others as not participating actively in discussion.*

Instead of dismissing comments that differed from their own views, many participants attempted to reconcile these contrasting perspectives. Students described becoming aware of previously unnoticed aspects of their behavior, particularly the visibility of their participation during discussion. They realized that intentions alone were insufficient if those intentions were not recognized by others.

*Through this peer evaluation, I could see myself more objectively. There were positive comments, but the areas for improvement were also clear, which gave me a great deal to think about.*

Several reflections suggested that peer evaluation functioned as a ‘social mirror.’ Participants described teammates’ comments as providing perspectives on their participation that differed from their own self-perceptions. This experience extended beyond performance appraisal and prompted reconsideration of what it meant to contribute effectively within a collaborative learning environment.

*I was proud that I had listened well and asked many questions, but if judged by how much I asserted my own opinion, the absolute amount may have been small.*

Collectively, these accounts described how peer evaluation could bring students’ self-perceptions into comparison with the perceptions reported by their peers.

### 3.4. Theme 3: Making Sense of Emotional Responses

Receiving peer evaluation was rarely described as an emotionally neutral experience. Participants reported feelings of anticipation before receiving feedback, followed by relief, pride, gratitude, disappointment, embarrassment, or discomfort depending on the nature of the comments. Students described these emotional reactions alongside efforts to interpret, evaluate, and make sense of the feedback they had received.

Students often described positive feedback in relation to confidence and behaviors they wished to maintain. Encouraging comments were frequently interpreted as recognition of their efforts by teammates and were accompanied by intentions to continue contributing actively.

*I often wondered whether my preparation was insufficient, but receiving confirmation through my teammates’ feedback put my mind somewhat at ease.*

Comments identifying areas for improvement were associated with varied responses. Some students described disappointment or discomfort and also considered whether the comment was specific, fair, or useful.

*Rather than seeing it as a negative comment, I will think of it as an opportunity to develop further and continue improving myself.*

The strongest emotional responses were described when comments were perceived as referring to personal characteristics rather than specific behaviors. Some students reported disappointment or hurt and also reflected on what they considered constructive, respectful, and educationally useful feedback.

*The feedback did not refer to a specific action I had taken; by saying that I “looked angry,” it felt like a sweeping judgment about me as a person, and I was deeply hurt.*

Across the journals, students described a range of emotional reactions and also reflected on the credibility and usefulness of the feedback they received. The present data do not establish a uniform sequence between emotion, appraisal, and subsequent action.

### 3.5. Theme 4: Transforming Feedback into Future Practice

Participants often wrote about how they intended to participate in future TBL sessions. These intentions included preparing more thoroughly, speaking up earlier, encouraging quieter peers, and contributing more actively during discussion. The journals document intended or anticipated changes rather than subsequent behavior.

*Whether my answer is right or wrong, I realized that I first have to open my mouth; only then can it help both me and my teammates.*

This statement was included in Theme 4 because it expresses an intention for future participation formed after receiving feedback, whereas Theme 1 concerns students’ descriptions of preparation before the evaluation. These reflections frequently contained specific intentions for future participation. They show how students described plans after receiving feedback; the present study did not examine whether these plans were later enacted.

*Even if I am wrong, I realized that expressing my opinion and sharing thoughts with teammates is more important than anything else.*

Several participants also broadened their goals beyond individual performance. They described intentions to facilitate discussion, invite diverse opinions, and support teammates who participated less actively, indicating an expanding understanding of collaborative responsibility.

*Because these are teammates I will continue working with, I resolved to remove the barriers as quickly as possible and contribute to the TBL team more naturally and actively.*

Although the specific intentions differed across participants, many students described feedback in relation to what they hoped to do differently in subsequent team learning.

Overall, participants expressed concrete intentions for future participation after receiving peer feedback.

### 3.6. Theme 5: Becoming a Responsible Evaluator

Although participants initially focused on receiving feedback, many reflections gradually shifted toward the experience of evaluating others. Students recognized that assigning scores and providing written comments required fairness, honesty, empathy, and professional judgment. The evaluator’s role was therefore experienced as a responsibility rather than a procedural requirement.

*Evaluating others was also difficult. Because everyone participated actively, it was hard to give someone a lower score.*

Participants frequently reflected on the ethical dimensions of peer evaluation. They questioned how to provide honest feedback without discouraging teammates and emphasized that comments should focus on observable behaviors rather than personal characteristics.

*When giving feedback about something the other person should improve, I learned that we should mention a concrete episode so that the recipient can understand and accept it.*

Several students reported that evaluating peers increased their awareness of how their own comments might influence others. Consequently, they described becoming more thoughtful when selecting words and framing suggestions for improvement.

*Knowing that all teammates had prepared and contributed actively, it was difficult to assign someone a below-average score. But when I considered the evaluation process itself as part of the team process, I resolved to give feedback as faithfully as possible.*

A small number of participants admitted feeling uncomfortable assigning lower scores to classmates despite recognizing meaningful differences in contribution. These accounts highlight the tension between maintaining interpersonal relationships and ensuring fairness within collaborative assessment.

Collectively, these reflections showed that some students reconsidered their responsibilities when evaluating peers, including fairness, specificity, empathy, and accountability.

### 3.7. The Essential Structure of Receiving Peer Evaluation

Across the journals, the five themes were often connected within the same reflection. Students wrote about their preparation and contribution, how they understood peers’ comments, their emotional responses, intentions for future participation, and their experience of evaluating others. These elements did not appear in a uniform order in every journal, and the present data do not establish a developmental sequence over time.

## 4. Discussion

This study explored the lived experience of receiving peer evaluation among preclinical medical students in a TBL-based course, using reflective journals completed after feedback was returned. Five interrelated themes described students’ preparation and contribution, perceptions of themselves through peers’ comments, emotional responses, intentions for future participation, and reflections on their role as evaluators. The following discussion offers theoretically informed interpretations of these descriptive findings. Feedback literacy and self-regulated learning are used as interpretive perspectives and should not be read as mechanisms demonstrated by the present data.

### 4.1. Preparing to Contribute: Accountability as the Entry Point of Feedback Uptake

Participants described pre-class preparation as related to their ability to contribute to team learning. This is consistent with the emphasis on individual accountability in TBL (Michaelsen et al., 2004; Cestone et al., 2008). From a feedback-literacy perspective, these accounts may also be read as showing that feedback had personal relevance because students were already invested in the collaborative activity being evaluated (Carless & Boud, 2018). This interpretation remains tentative because the study did not examine feedback uptake prospectively.

### 4.2. Encountering Oneself Through Peers’ Eyes: Feedback as Informed Self-Assessment

Participants’ accounts often contrasted their own intentions with how they believed teammates had perceived their behavior. This is consistent with literature emphasizing the limits of unaided self-assessment and the value of external information for calibrating self-judgment (Eva & Regehr, 2005; Sargeant et al., 2010). The present findings describe occasions on which peer comments prompted reconsideration of self-perception; they do not establish that peer evaluation improved self-assessment accuracy.

### 4.3. Making Sense of Emotional Responses: Managing Affect and Judging Credibility

Emotional responses were part of students’ accounts of receiving peer evaluation, ranging from pride and gratitude to disappointment and hurt. Students also described judgments about the credibility or usefulness of the comments they received. In some accounts these elements appeared together, whereas in others discomfort remained evident. Behaviorally specific comments were often described as easier to consider than comments perceived as person-focused. These observations are consistent with the managing-affect dimension of feedback literacy (Carless & Boud, 2018; Sargeant et al., 2008) and with research on credibility judgments in feedback (Watling et al., 2012; Telio et al., 2015), but the present data do not establish a causal or uniform temporal sequence between emotion and appraisal. The findings should also be considered in relation to the South Korean educational context. Previous research has described cultural variation in norms surrounding interpersonal harmony, criticism, and participation (Frambach et al., 2012); however, cultural influences were not examined directly in this study and the participants’ responses should not be attributed to cultural norms on the basis of these data alone.

### 4.4. Transforming Feedback into Future Practice

Many participants expressed intentions for future TBL sessions, such as preparing more thoroughly, speaking up earlier, or encouraging quieter teammates. Viewed through Zimmerman’s model of self-regulated learning (Zimmerman, 2002), these expressed intentions may be understood as consistent with movement from reflection toward future planning. However, the journals document intentions rather than subsequent behavior, and the study did not examine whether these plans were enacted. Similarly, Boud and Molloy’s concept of sustainable feedback (Boud & Molloy, 2013) provides a useful perspective for considering future-oriented use of feedback, but the present data do not demonstrate an effect on later performance.

### 4.5. Becoming a Responsible Evaluator: Reciprocity and Emerging Professionalism

The final theme concerned students’ reflections on their own role as evaluators. Some described discomfort when differentiating scores among teammates and articulated standards they considered important for feedback, including fairness, specificity, behavioral focus, and an orientation toward improvement. These accounts are consistent with prior research showing that students may find constructive peer criticism difficult (Burgess et al., 2021; Lerchenfeldt et al., 2023). They also suggest occasions on which receiving feedback prompted students to reconsider how they themselves evaluated peers. Whether repeated experience in both roles contributes to evaluative judgment or professional responsibility requires further study (Tai et al., 2018; Boud & Soler, 2016).

### 4.6. Implications for Practice

The five themes can be considered in relation to dimensions of student feedback literacy, including appreciating feedback, making judgments, managing affect, and taking action (Carless & Boud, 2018; Molloy et al., 2020). This comparison was made after completion of the inductive phenomenological analysis and is offered as an interpretive lens rather than as evidence that feedback literacy developed during the course. Theme 5 also raises questions about how receiving and giving feedback may be related, which could be examined in longitudinal studies.

Several educational considerations arise from these accounts. Educators using peer evaluation may wish to provide guidance on behaviorally specific comments and to allow students an opportunity to reflect after receiving feedback. The open-ended reflection used in this course provided students with space to describe their reactions, interpretations, and intentions, but the present design cannot determine whether reflection itself changed feedback uptake or subsequent behavior. The evaluator role may also be discussed explicitly, particularly where scoring procedures require students to differentiate among teammates. These implications should therefore be viewed as considerations for educational design and future study rather than as effects demonstrated by the present study.

### 4.7. Strengths, Limitations, and Future Directions

A strength of this study is that the reflective journals were completed close to the time students received feedback and the dataset included the available course cohort. Several limitations should also be acknowledged. The study involved one cohort at one medical school in South Korea, which limits transferability. The journals were completed as a required course activity, which may have influenced what students chose to write. Participant validation, the seventh step in Colaizzi’s original procedure, was not conducted because archived data were analyzed; consequently, we could not determine whether participants would recognize the synthesized description as reflecting their experience. Translation of selected quotations from Korean to English may also have altered nuance. Because reflective writing was both part of the post-feedback activity and the data source, the study cannot distinguish experiences prompted by receiving peer feedback from those elicited or shaped by the act of reflection itself. The journals document intentions rather than enacted behavior. In addition, demographic characteristics and TBL performance data were not linked to individual de-identified journals, so gender-specific analyses and analyses relating the reflections to iRAT, tRAT, or application-exercise performance were not possible.

Future studies could examine whether stated intentions are enacted in subsequent team behavior through longitudinal designs linking reflections with observed participation or later peer-evaluation outcomes. Comparative studies across institutions and collaborative learning formats could examine the transferability of the findings. Studies that retain appropriate links between de-identified demographic data, performance measures, and reflective texts could explore possible variation by gender or other characteristics (Lin et al., 2016) and relationships with iRAT, tRAT, or application-exercise performance. Comparative designs could also examine whether experiences differ when peer evaluation is or is not followed by reflection.

## 5. Conclusions

For these preclinical medical students, reflective accounts after peer evaluation included self-assessment, emotional responses, judgments about feedback, intentions for future participation, and reflections on evaluating peers. The findings draw attention to the experience of receiving peer feedback, in addition to the quality of feedback provided. Pairing peer evaluation with an opportunity for reflection may provide a useful context for examining how students interpret and respond to feedback. Whether this approach contributes to subsequent behavior, feedback literacy, self-regulated learning, or professionalism requires further longitudinal and comparative research.

## Figures and Tables

**Table 1:** Themes describing the lived experience of receiving peer evaluation, with representative quotations.

Theme	Essence of the Experience	Representative Quotation
1. Preparing to contribute	Preparation was experienced as readiness to contribute to the team and as accountability toward peers, rather than as an individual academic activity.	“I tried to prepare thoroughly both for myself and to avoid causing difficulty for my teammates.”
2. Encountering oneself through peers’ eyes	Peer feedback was described as a social mirror that brought students’ self-perceptions into comparison with peers’ perceptions.	“Through this peer evaluation, I could see myself more objectively.”
3. Making sense of emotional responses	Students described emotional responses alongside judgments about the credibility and usefulness of the feedback they received.	“Rather than seeing it as a negative comment, I will think of it as an opportunity to develop further and continue improving myself.”
4. Transforming feedback into future practice	Students expressed intentions to adjust their preparation and participation in future team learning.	“Whether my answer is right or wrong, I realized that I first have to open my mouth; only then can it help both me and my teammates.”
5. Becoming a responsible evaluator	Students reflected on their responsibilities as evaluators, including fairness, specificity, empathy, and accountability.	“When giving feedback about something the other person should improve, I learned that we should mention a concrete episode so that the recipient can understand and accept it.”

## Data Availability

The data that support the findings of this study are not publicly available because they contain information that could compromise the privacy of student participants. De-identified data are available from the corresponding author upon reasonable request.

## Supplementary Materials

The following supporting information can be downloaded at: https://www.mdpi.com/article/10.3390/bs16091462/s1, S1: Peer-evaluation form used in the TBL course.
