# Supplementary material for: Receiving Peer Evaluation in Team-Based Learning: A Descriptive Phenomenological Study of Preclinical Medical Students
Source: Behav Sci (Basel). 2026 Aug 23;16(9):1462. doi: 10.3390/bs16091462 (PMC13603606; doi:10.3390/bs16091462)
Supplement: Supplementary file 1 [file behavsci-16-01462-s001.zip › behavsci-4473575-supplementary.pdf]

Supplementary Material S1: Peer-evaluation form used in the TBL course.

The original form was administered in Korean. Each student completed one form, rating every other member of their team.

Evaluation domains

Domain	Guiding question / description
Preparedness	Did the teammate prepare thoroughly for class through pre-class study?
Team contribution	Active participation; discussion based on evidence; cooperation with, and leadership of, team members
Consideration and respect	Did the teammate treat fellow team members with consideration and respect?

Scoring rules

- Scores were assigned so that the average score per teammate was 10 points.
- Each evaluator was required to assign a score of 9 or below to exactly one teammate and a score of 11 or above to exactly one teammate.

Rating and rationale grid (completed for each teammate)

Teammate (name)	Preparedness (10)	Team contribution (10)	Consideration & respect (10)	Rationale for the evaluation

Note. A written rationale was mandatory for the teammates to whom the evaluator assigned the highest and the lowest scores.
